# Intra-amniotic Sildenafil Treatment Modulates Vascular Smooth Muscle Cell Phenotype in the Nitrofen Model of Congenital Diaphragmatic Hernia

**DOI:** 10.1038/s41598-018-34948-w

**Published:** 2018-12-05

**Authors:** Frances C. Okolo, Guangfeng Zhang, Julie Rhodes, Douglas A. Potoka

**Affiliations:** 1 Department of Surgery, University of Pittsburgh Medical Center, Pittsburgh, PA USA; 2 Department of Pediatric General and Thoracic Surgery, Children’s Hospital of Pittsburgh of University of Pittsburgh Medical Center, Pittsburgh, PA USA

## Abstract

The etiology of pulmonary vascular abnormalities in CDH is incompletely understood. Studies have demonstrated improvement in pulmonary vasculature with prenatal therapy in animal models. We hypothesize that prenatal sildenafil may attenuate defective pulmonary vascular development via modulation of vSMC phenotype from undifferentiated, proliferative phenotype to differentiated, contractile phenotype. We utilized the nitrofen model of CDH to examine the effect of IA sildenafil on pulmonary vSMC phenotype during lung development. Timed-pregnant CD-1 mice were gavage fed 25 mg nitrofen or olive oil (control) at E8.5 of gestation. Single IA injections of Sildenafil (Revatio; 10 µL of 4 mg/4 ml solution) or dextrose control were performed at E12.5. Mice were sacrificed on various gestational days for embryonic lung harvest. Markers of vSMC development of undifferentiated and differentiated phenotypes were analyzed by immunostaining and western blot. Across all time points in gestation, nitrofen-treated embryonic lungs demonstrated increased vSMC expression of NOTCH3, Hes-5, PDGFR-β, desmin and α-SMA and decreased expression of calponin and SMMHC, compared to oil controls. IA dextrose treatment had no effect on expression levels. However, IA Sildenafil treatment resulted in down-regulation of NOTCH3, Hes-5, PDGFR-β, desmin and α-SMA and upregulation of calponin and SMMHC, comparable to oil controls. In the nitrofen model, vSMC express markers consistent with more undifferentiated proliferative phenotype, resulting in hypermuscularization of intrapulmonary arterioles in CDH. A single dose of IA Sildenafil treatment early in gestation, results in sustained normalization of vSMC phenotype. Pharmacologic modulation of the vSMC phenotype at key gestational points may have therapeutic potential.

## Introduction

Congenital diaphragmatic hernia (CDH) is a relatively common birth defect that is associated with significant mortality and morbidity^1,2^. Postnatal clinical instability and mortality associated with CDH is mainly due to lung maldevelopment, resulting in pulmonary hypertension and often severe respiratory failure in newborns with CDH^3^. These pulmonary developmental defects include lung hypoplasia and overly muscularized distal pulmonary arterioles, with increased medial and adventitial wall thickness and decreased pulmonary arterial cross-section^4–6^. The increased levels of α-SMA per vessel seen in these hypoplastic lungs on fetal studies further demonstrates the hypermuscularization of these intra-acinar vessels^7,8^. There is increasing evidence that these pulmonary anomalies are not solely secondary to the mechanical compression of the lung from herniation of intra-abdominal contents. Gene mutations such as FOG2, abnormalities in the retinoic acid pathway and the “dual-hit hypothesis” have all been implicated in CDH associated pulmonary hypoplasia^9–12^. Advancements in neonatal respiratory care, such as inhaled nitric oxide, have not resulted in an increase in survival rates in subsets of patients with CDH^13,14^. The inability of these therapies to improve outcomes suggests that a fixed component of the defective pulmonary vasculature is not responsive to postnatal pulmonary vasodilator therapy^15,16^ and that directed prenatal therapy targeting the developing pulmonary vasculature may improve outcomes in CDH.

Several existing studies have explored the use of prenatal drug therapy to modulate the developing pulmonary vasculature in animal models of CDH. Chang *et al*. utilized oral maternal imatinib, a PDGFR antagonist, in the rat nitrofen model, revealing significantly decreased medial wall thickness in pulmonary arteries as well as downregulation of PDGFR-β which had been upregulated in the nitrofen model^17^. Another study by Makanga *et al*. demonstrated similar effects following oral maternal simvastatin in the rat nitrofen model^18^. There have been several studies with sildenafil demonstrating similar effects. Luong *et al*. administered maternal sildenafil (100 mg/kg/d subcutaneously) from E11.5 to E20.5 in the rat nitrofen model and evaluated fetal lungs just prior to term. In sildenafil-treated lungs, there was increased pulmonary vessel density, increased eNOS and VEGF expression, an enhanced vasodilatory response to NO, and decreased right ventricular hypertrophy^19^. A similar study by Burgos *et al*. performed daily subcutaneous sildenafil treatments at the same dose and same gestational periods and demonstrated decreased PA resistance and improved oxygenation^20^. There is only one study known to date that has evaluated sildenafil administration somewhat directly to the fetus. Daily transplacental sildenafil treatments (10 mg/kg/day) performed in the rabbit nitrofen model of CDH by Russo *et al*. from GD24 to term (GD30) resulted in normal medial and adventitial thickness in peripheral pulmonary vessels with normal vascular branching and reduction in pulmonary vascular resistance without obvious fetal or maternal toxicity^21^.

We sought to determine the mechanisms by which prenatal treatment with sildenafil may modulate vascular smooth muscle cells (vSMC) within the developing pulmonary vasculature in CDH. We hypothesize that prenatal sildenafil may attenuate defective pulmonary vascular development in CDH via modulation of vSMC phenotype. vSMC can exist along a continuum of phenotypes ranging from a relatively undifferentiated, proliferative phenotype, which express higher levels of PDGFR-β and Notch3 to a more fully differentiated contractile phenotype expressing higher levels of cyclic GMP dependent protein kinase (PKG), calponin, and smooth muscle myosin heavy chain (SMMHC)^22,23^. Notch and PDGF signaling are two of the most important signaling pathways associated with endothelial cell differentiation. Notch3, expressed in vSMC, acts upstream of PDGF signaling and its receptor PDGFR-β^24,25^. Notch3 deficient mice demonstrated reduced PDGFR-β expression as well as structural defects and impaired vSMC differentiation in the distal arteries^26^. SMMHC and calponin are expressed relatively late in vSMC differentiation^27–29^. Defective vSMC differentiation resulting in a shift to a more immature, proliferative vSMC phenotype at key stages of pulmonary vascular development could theoretically lead to hypermuscularization of pulmonary arterioles as seen in CDH. Thus, we sought to investigate the pattern of expression of these markers of vSMC phenotype and the effects of prenatal intra-amniotic (IA) sildenafil treatment on pulmonary vSMC phenotype during lung development in the murine nitrofen model of CDH.

## Results

**Figure 1:**
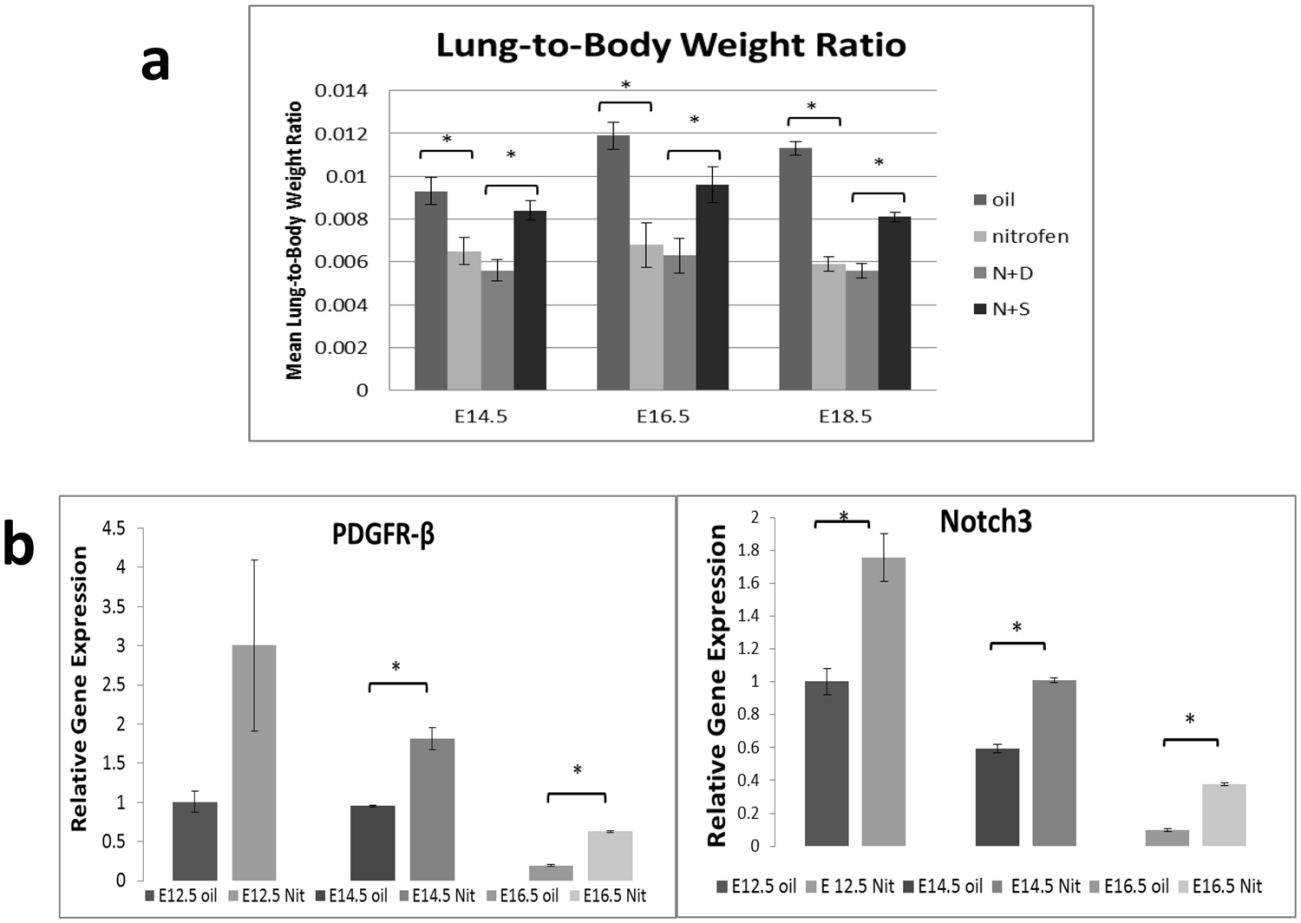
(**a**) Mean lung weight to body weight ratio at varying time points in gestation. The lung to body weight ratio in the oil group at all time points in gestation were significantly higher than in the nitrofen group. IA dextrose treatment of the nitrofen treated pups had no effect on the lung or body weight. Nitrofen treated pups exhibited higher lung-to-body weight ratio approximating oil controls following IA Sildenafil treatment. Data were analyzed using one-way ANOVA with post-hoc Tukey HSD test. Significant p values are shown (* p < 0.05). (**b**) Relative gene expression of PDGFR-β and Notch 3 in oil and nitrofen groups at varying time points in gestation by quantitative PCR. At all time points in gestation, PDGFR-β and Notch 3 demonstrate increased relative expression in the nitrofen treated lungs as compared to oil group. Statistical analysis was performed using Student’s t-test. Significant p values are shown (*p < 0.05).

In nitrofen-treated embryos, approximately 50–70% of pups were noted to have a diaphragmatic defect, although all nitrofen-treated embryonic lungs demonstrated similar degrees of lung hypoplasia and mesenchymal PDGFR-β immunostaining regardless of the presence of a diaphragmatic defect, as confirmed on histological analysis of left lobes of the lung (Supplementary Fig. 1). This was also evident in the decrease in the left lung weight to body weight ratio at E14.5, E16.5 and E18.5. (Fig. 1a). Intra-amniotic sildenafil treatment significantly increased left lung weight to body weight ratio in nitrofen-treated embryos while intra-amniotic dextrose had no effect on the weights of nitrofen-treated embryos or lungs. At E12.5, E14.5 and E16.5, there was a consistent pattern of increased gene expression of Notch3 and PDGFR-β in nitrofen-treated lungs compared to oil controls by RT-PCR (Fig. 1b), suggesting a more immature and proliferative vSMC phenotype in nitrofen-treated embryonic lungs.

**Figure 2:**
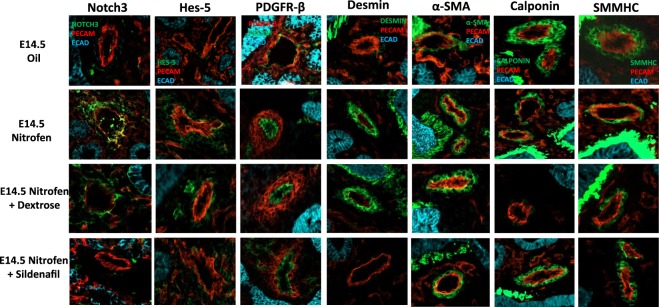
Immunofluorescent Staining patterns of the left lobe of the lung at E14.5 with antibodies against CD31 (endothelial cells), ECAD (epithelial cells) and various markers of VSMC differentiation in oil, nitrofen, nitrofen plus IA dextrose and nitrofen plus IA Sildenafil groups. At E14.5, the expression of Notch3, Hes5, PDGFR-β, Desmin and α-SMA is decreased while the expression of calponin and SMMHC is increased in the nitrofen groups with no change following IA dextrose treatment. Following IA Sildenafil treatment, vSMC expression of these markers normalizes comparable to that seen in the oil control group.

**Figure 3:**
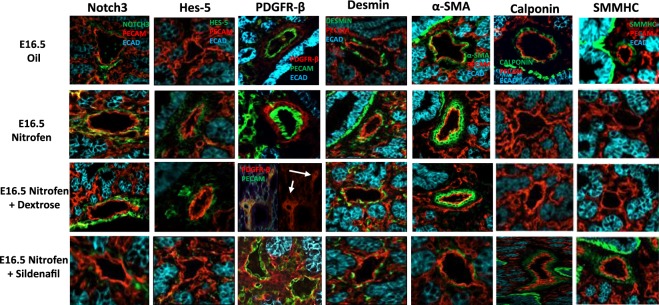
Immunofluorescent Staining patterns of the left lobe of the lung at E16.5 with antibodies against CD31 (endothelial cells), ECAD (epithelial cells) and various markers of VSMC differentiation in oil, nitrofen, nitrofen plus dextrose and nitrofen plus Sildenafil groups. At E16.5, The expression of Notch3, Hes5, PDGFR-β, Desmin and α-SMA is decreased while the expression of calponin and SMMHC is increased in the nitrofen groups with no change following IA dextrose treatment. Following IA Sildenafil treatment, vSMC expression of these markers normalizes comparable to that seen in the oil control group.

**Figure 4:**
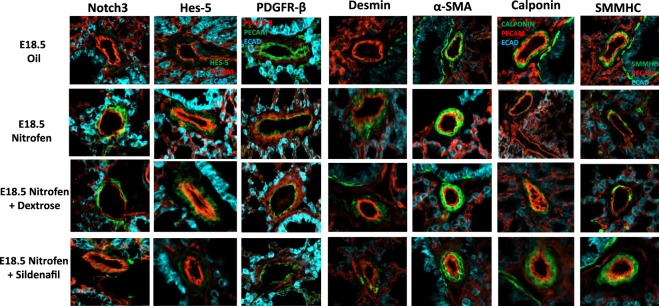
Immunofluorescent Staining patterns of the left lobe of the lung at E18.5 with antibodies against CD31 (endothelial cells), ECAD (epithelial cells) and various markers of VSMC differentiation in oil, nitrofen, nitrofen plus dextrose and nitrofen plus Sildenafil groups. At E18.5, The expression of Notch3, Hes5, PDGFR-β, Desmin and α-SMA is decreased while the expression of calponin and SMMHC is increased in the nitrofen groups with no change following IA dextrose treatment. Following IA Sildenafil treatment, vSMC expression of these markers normalizes comparable to that seen in the oil control group.

**Figure 5:**
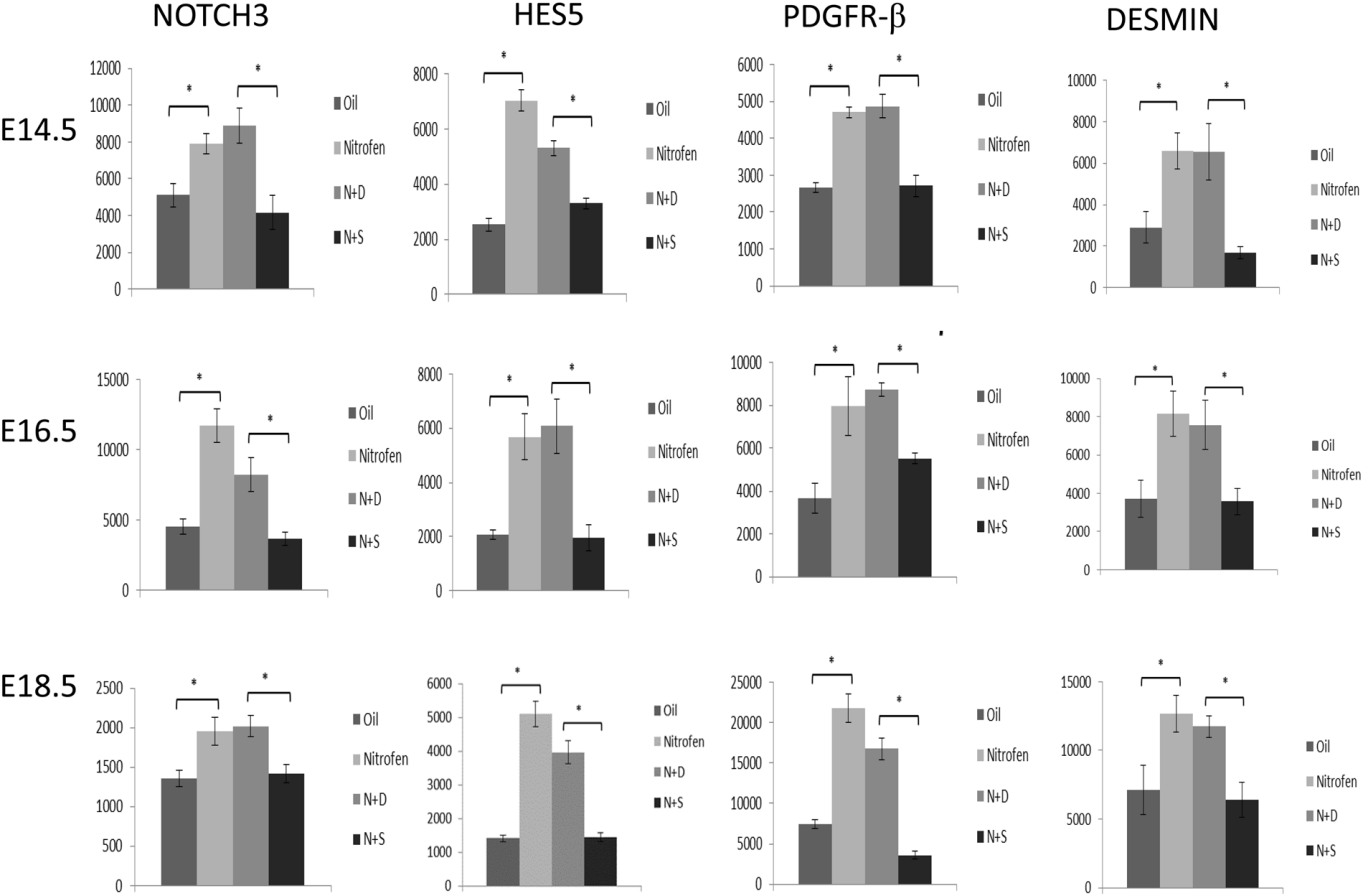
CTCF analysis of fluorescence intensity of undifferentiated markers of vascular smooth muscle development in oil, nitrofen, nitrofen plus IA dextrose and nitrofen plus IA Sildenafil groups at E14.5, E16.5 and E18.5. Data are mean ± SEM of fluorescence intensity of vSMCs surrounding small pulmonary arterioles. Data were analyzed using One-Way ANOVA with post-hoc Tukey HSD test. Significant p values are shown (*p < 0.05).

**Figure 6:**
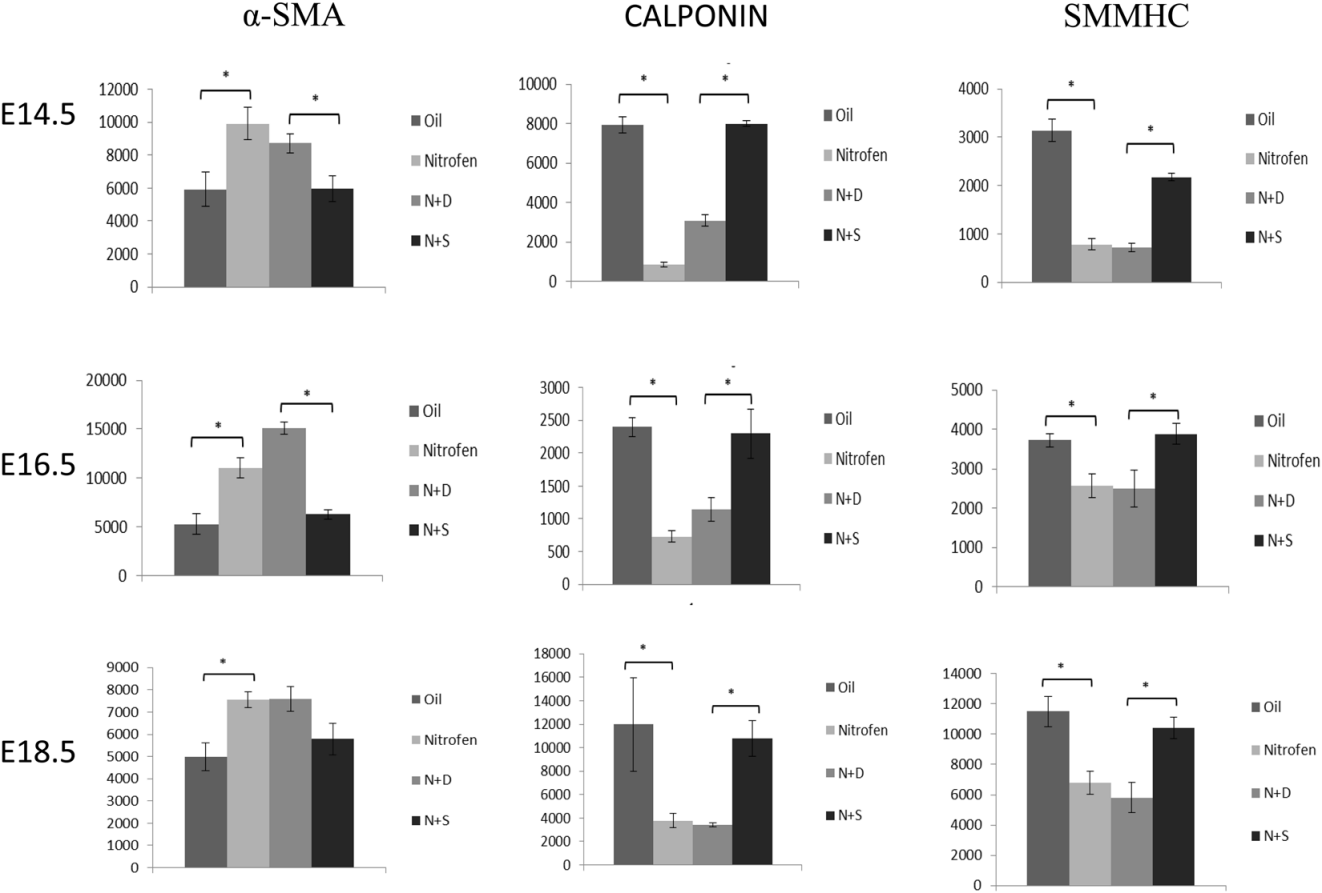
CTCF analysis of fluorescence intensity of differentiated markers of vascular smooth muscle development in oil, nitrofen, nitrofen plus IA dextrose and nitrofen plus IA Sildenafil groups at E14.5, E16.5 and E18.5. Data are mean ± SEM of fluorescence intensity of vSMCs surrounding small pulmonary arterioles. Data were analyzed using One-Way ANOVA with post-hoc Tukey HSD test. Significant p values are shown (*p < 0.05).

**Figure 7:**
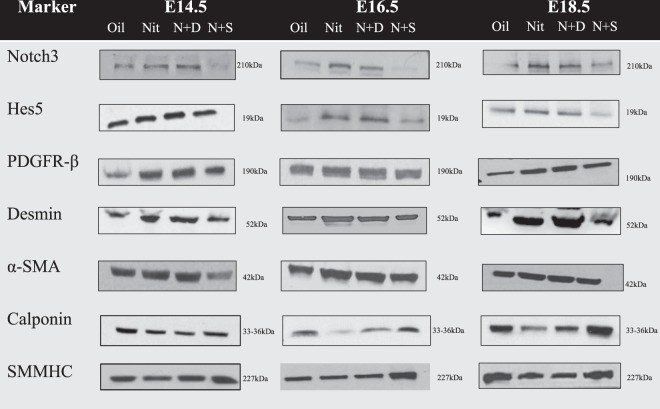
Protein expression levels of various markers of vSMC differentiation in oil, nitrofen, nitrofen plus IA dextrose and nitrofen plus IA sildenafil groups. Lungs were harvested from treated pups at E14.5, E16.5, and E18.5 for protein preparation. Protein samples were analyzed by Western blot. Beta-actin was used as an internal standard.

**Figure 8:**
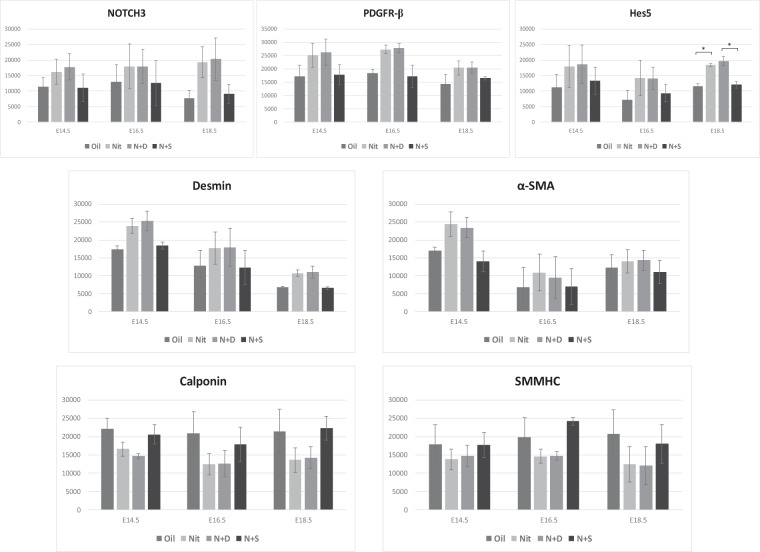
Quantification of western blots of various markers of vSMC differentiation in oil, nitrofen, nitrofen plus IA dextrose and nitrofen plus IA sildenafil groups. Data are mean ± SEM of band density. Data were analyzed using One-Way ANOVA with post-hoc Tukey HSD test. Significant p values are shown (*p < 0.05).

We next examined the expression pattern of the vSMC markers (Notch3, Hes-5, PDGFR-β, desmin, α-SMA, calponin, and SMMHC) within the lungs of oil and nitrofen-treated embryos, as well as nitrofen-treated embryos subjected to intra-amniotic dextrose control or intra-amniotic sildenafil. At E14.5 (Fig. 2), E16.5 (Fig. 3), and E18.5 (Fig. 4), there was a consistent pattern with increased perivascular expression of Notch3, Hes-5, PDGFR-β, desmin and α-SMA and decreased expression of calponin and SMMHC in the nitrofen-treated lungs compared to oil controls. Intra-amniotic injection of dextrose had no effect on vSMC marker expression within the nitrofen group, while intra-amniotic sildenafil injection normalized expression with decreased perivascular expression of Notch3, Hes-5, PDGFR-β, desmin, α-SMA, and increased expression of calponin and SMMHC compared to the nitrofen and nitrofen plus intra-amniotic dextrose groups at all time points. These findings were confirmed and determined to be significant via quantification of immunofluorescence by determining the CTCF (Figs 5, 6). To further validate the results from immunohistochemistry, western blot analysis for the various vSMC markers was performed (Figs 7, 8). Western blot once again confirmed the overall trend of increased expression of Notch3, Hes-5, PDGFR-β, desmin, and α-SMA and decreased expression of calponin and SMMHC in the nitrofen-treated lungs compared to oil controls at E14.5, E16.5, and E18.5. Intra-amniotic dextrose treatment had no effect on protein expression patterns compared to the nitrofen group, while intra-amniotic sildenafil treatment normalized protein expression to patterns similar to oil controls. Taken together, these results suggest that in nitrofen-treated lungs, there is a shift to a more undifferentiated, proliferative vSMC phenotype at the gestational time points examined, and that intra-amniotic sildenafil promotes normal vSMC differentiation in nitrofen-treated lungs.

## Discussion

Lung hypoplasia is one of the key anatomic anomalies noted in human CDH. Here, we show that nitrofen treatment leads to lung hypoplasia. The lungs are significantly smaller in size in nitrofen treated pups with and without a diaphragmatic defect compared to controls. In addition, the body weights of the nitrofen treated pups are decreased however, this decrease is disproportionate to the decrease in lung weight as depicted in our results. The etiology of the lung hypoplasia is unlikely to be secondary to herniation of intra-abdominal contents. In mice that developed a diaphragmatic hernia defect following nitrofen treatment, there was an absence of intra-thoracic herniation (image not shown). Additionally, these pups are treated with sildenafil in utero prior to a diagnosis of CDH and improvements in lung hypoplasia are demonstrated both in pups with and without a physical diaphragmatic hernia.

Changes in the signaling pathways associated with vSMC phenotype have been implicated in cardiovascular disease from systemic hypertension to atheroma plaque and aneurysm formation^30–32^. We propose that alterations of vSMC phenotype at key stages of pulmonary vascular development may contribute to hypermuscularization of pulmonary arterioles in CDH and that vSMC phenotype could be modulated by prenatal drug therapy. Markers of differentiated vSMC include components of the contractile apparatus, such as calponin, SMMHC, α-SMA, and desmin^22,23,28,33^ while undifferentiated, proliferating vSMC during development express higher levels of PDGFR-β and Notch3^24^. Presumably, vSMC differentiation is regulated during lung development to maintain normal vascular wall maturation of the pulmonary vasculature.

We chose to evaluate gene expression of Notch3 and PDGFR-β because these are some of the key signaling pathways in vSMC development and differentiation as discussed above. Differences in expression of these genes between the oil and nitrofen groups would further confirm similarities between the nitrofen model of CDH and human CDH. As expected, we found an increase in Notch3 and PDGFR-β gene expression via RT-PCR in nitrofen treated lungs across all time points in gestation. Morrow *et al*. have demonstrated that Notch3 signaling through Hes-5 is associated with vSMC proliferation and a shift to an undifferentiated, more proliferative vSMC phenotype^34^. Therefore, it appears that in the nitrofen group, there is a delay in vSMC differentiation leading to over-proliferation of vSMC, which is likely to contribute to hypermuscularization of pulmonary arterioles.

At all time-points analyzed, nitrofen treatment resulted in a consistent pattern of alteration of expression of markers of vSMC phenotype. Specifically, compared to oil controls, nitrofen-treated embryonic lungs exhibited decreased expression of calponin and SMMHC and increased expression of Notch3, Hes5, and PDGFR-β. Calponin and SMMHC are later markers of more differentiated vSMC, while desmin and α-SMA are relatively earlier markers of vSMC differentiation^28,35–39^. Thus, the decreased expression of calponin and SMMHC and increased expression of Notch3, Hes5, and PDGFR-β once again supports a shift towards a less differentiated, more proliferative vSMC phenotype potentially contributing to hypermuscularization of the pulmonary vasculature in CDH. Since desmin and α-SMA are earlier markers of vSMC differentiation, the increased expression of these markers in the nitrofen group may either be due to the presence of an intermediate vSMC phenotype or to an increase of vSMC mass in the nitrofen-treated lungs. Of note, Sluiter *et al*. demonstrated hypoxia-induced expression of α-SMA in the most distal vessels which normally are non-muscularized, suggesting possible differentiation of these cells into smooth muscle or smooth muscle-like cells in periods of hypoxia^37^.

Although our results reveal a modulation of vSMC phenotype in the nitrofen model with the use of intra-amniotic (IA) sildenafil, there are limitations to this study. IA Sildenafil treatment was performed at E12.5, which corresponds to 6^th^ week of gestation in humans. Human CDH can be detected on ultrasound as early as 14 to 16 weeks of gestation which makes our IA sildenafil treatment timing premature^40^. However, these results do suggest that prenatal vasodilator therapy can modulate vSMC phenotype within the developing lung and therefore deserves further study to clarify its therapeutic potential. In addition, translation to human trials would require amniocentesis, a relatively safe procedure which carries a low risk of complications with more recent literature suggesting a risk of miscarriage of 0.1%^41^.

In conclusion, our results suggest that vSMC phenotype can be modulated by prenatal drug therapy. Specifically, in nitrofen-treated embryonic lungs, a single dose of IA sildenafil treatment resulted in a sustained normalization of the pattern of expression of markers of vSMC phenotype resulting in a pattern similar to oil controls, while IA dextrose control had no effect. The etiology of the alterations in vSMC phenotype seen with nitrofen treatment is unknown; however, the remarkable attenuation of these changes seen in the nitrofen model of CDH following a single dose of IA Sildenafil suggests that the nitric oxide signaling pathway is involved. Sildenafil inhibits phosphodiesterase type 5 (PDE5) which results in an increase in cGMP levels and upregulation of activity of Protein kinase G (PKG), a cGMP dependent protein kinase. In vSMC, PKG inhibits proliferation and promotes differentiation. Additional studies are necessary to further elucidate the underlying mechanisms involved in the altered vSMC seen in the nitrofen model and the sustained effect of IA sildenafil on vSMC phenotype, and to correlate these findings to the human disease. Our results and those of others, suggest that prenatal vasodilator therapy may hold some promise for therapy for CDH. We have utilized intra-amniotic administration of sildenafil, which may result in higher concentrations or more sustained drug delivery to the embryo without maternal effects, although the pharmacokinetics of the mode of delivery and the mechanisms responsible for the sustained effect on vSMC phenotype following a single treatment of IA sildenafil need to be further investigated.

## Materials and Methods

Timed-pregnant CD1 mice were obtained from Charles Rivers Laboratories (Wilmington, MA). Timing of pregnancies was determined from the appearance of a vaginal plug, designated as (E) 0.5. At embryonic day (E) 8.5, pregnant females were gavage fed 25 mg of nitrofen (Wako Chemicals) completely dissolved in 1 mL of olive oil. Controls were fed 1 mL of olive oil alone. At E12.5, pregnant females were temporarily anesthetized using isoflurane and a laparotomy was performed to access the fetuses using sterile surgical techniques. A single dose of sildenafil (10 µL of 4 mg/4 ml solution per embryo) or dextrose (control) was injected under direct visualization into the amniotic fluid of the fetuses. The longitudinal incision was subsequently closed with 4-0 vicryl sutures. Pregnancy was continued and mice were sacrificed at E14.5, E16.5, and E18.5 by carbon dioxide (CO_2_) euthanasia. The fetuses were delivered by caesarean section and decapitated. Embryonic left lungs were harvested for sectioning and protein extraction. Expression of markers associated with undifferentiated and differentiated vSMC were analyzed using immunofluorescent staining and western blot.

Notch3 and PDGFR-β gene expression in oil and nitrofen-treated embryonic lungs was determined via RT-PCR. In this case, sildenafil or dextrose was injected into the amniotic fluid of the fetuses at E10.5 and gene expression of Notch3 and PDGFR-β were evaluated at E12.5, E14.5 and E16.5. Each of these experiments was performed with 1–2 litters per group per experiment. Each litter consisted of an average of 8 ± 3 pups (Mean ± S.D.). All experimental protocols and methods were approved and carried out in accordance with guidelines and regulations set by The University of Pittsburgh Institutional Animal Care and Use Committee (IACUC). All materials and data are available without restriction.

### Sildenafil Dosing

Dosing of Sildenafil was chosen at random. The dose of Sildenafil used was determined based on the drug concentration and amount of drug that was injectable.

### Real-Time Quantitative Polymerase Chain Reaction

Isolation of mRNA and reverse transcription into cDNA were performed using the µMACS One Step cDNA kit (Miltenyi Biotec). We utilized pre-designed primer assays (QuantiTect Primer Assay, Qiagen) which contain a lyophilized mix of forward and reverse primers corresponding to the genes of interest (Notch3, PDGFR-β). qPCR analysis was then performed using PerfeCTa SYBR Green SuperMix for iQ (Quanta Biosciences) and gene expression was measured by real-time PCR using iCycler iQ Real-Time PCR detection system (Bio-Rad).

Real-time PCR results obtained were calculated via relative quantification using gene expression levels in oil groups as a reference. The expression level of Glyceraldehyde 3-phosphate dehydrogenase (GAPDH) was utilized as an internal control. Statistical analyses of results were performed using the Student’s t-test.

### Immunohistochemistry

Fetal lungs were washed in PBS then fixed in 4% paraformaldehyde (PFA) at 4 °C for 16–24 hours. Specimens were cryoprotected in 30% sucrose for the same amount of time then embedded in a cassette filled with Tissue-Tek Optimal Cutting Temperature (O.C.T) embedding compound. Cassettes containing specimen were then flash frozen in liquid nitrogen and sectioned using a cryostat with thickness set to 6 µm. Sections were then mounted on slides with 6–8 sections per slide, labeled appropriately and stored at −20 °C until ready for staining. Seven primary antibodies were used including desmin, calponin, smooth muscle myosin heavy chain (SMMHC), smooth muscle actin (SMA), Hes-5, Notch3 and platelet derived growth factor β (PDGFR-β) (Supplementary Table 1). Sections were blocked in 10% NDS for 1 hour then incubated overnight at 4 °C in appropriate concentrations of primary antibody. In order to differentiate epithelium from vascular endothelium, Anti-E cadherin antibody (Anti-ECAD), expressed by epithelial cells and Anti-CD31 (PECAM), highly expressed in endothelial cells, were stained against the primary antibodies in question (Supplementary Table 1). After three washes with PBS and PBS-T, sections were incubated in a 1:200 dilution of different fluorophore-conjugated secondary antibodies from Jackson ImmunoResearch (Cy2, Cy3 or Cy5) for 1 hour at room temperature (20–25 °C). After incubation, the slides were once again washed in PBS, and coverslips were adhered to slides using Aqua Poly/Mount (Polysciences, Inc.) and stored horizontally at 4 °C with minimal light exposure until ready for imaging. Slides were imaged using Zeiss AxioImager Z1 and the Volocity 6.3 imaging software. Pulmonary vessels were identified by the presence of PECAM while the epithelium was identified by the presence of ECAD. Images were captured under 10X and 20X objectives with focus on arterioles. Cell fluorescence of these same images was then quantified using ImageJ to obtain mean corrected total cell fluorescence (CTCF) of 6–8 readings/areas per high power field. The software allowed exclusion of the airways thus solely readings of blood vessels in the images were used to calculate CTCF. Box plots were derived using Microsoft Office Excel while statistical analyses (Analysis of Variance in conjunction with Tukey HSD test) were performed using GraphPad Prism 7.

### Hematoxylin and Eosin (H&E) staining

Previously stored lung sections (as described in the immunochemistry section above) were allowed to thaw and air dry for several minutes prior to staining. Staining was performed as follows: hematoxylin dye for 1 minute, distilled water for 2 mins, eosin dye for 30 seconds, 70% ethanol for 30 secs, 80% ethanol for 30 secs, 90% ethanol for 30 secs, 95% ethanol for 30 secs, 100% ethanol for 30 secs (twice), xylene for 15 mins (twice). Histomount was then used for mounting and the slides were imaged using Nikon Eclipse E800 and the NIS-Elements D microscope imaging software. Images were captured under 5X objective.

### Western Blot

Protein levels from lung tissue lysates were measured using BSA protein assay. Appropriate amounts of protein from each group (oil, nitrofen, nitrofen + dextrose, nitrofen + sildenafil) were loaded on 4–20% Mini-PROTEAN TGX Precast gels (Bio-Rad) and run using standard methods. Proteins were then transferred to 0.2 µm PVDF membranes (Bio-Rad) and incubated with the indicated primary antibodies overnight at 4 °C (Supplementary Table 2). Next, the blots were incubated with the appropriate anti-IgG secondary antibodies conjugated with horseradish peroxidase (Dako) for 1 hour at room temperature (Supplementary Table 2). Protein bands were detected through autoradiography using HyGlo chemiluminescent detection reagent (Denville). Blots were checked for equal loading by re-probing with anti-β actin. All sample comparisons were performed on the same gel. Blots were quantified using ImageJ and graphed using Microsoft Office Excel. Blots were physically cropped at respective molecular weights before incubating with primary antibody for the purposes of probing several antibodies simultaneously.

### Statistical Analyses

Mean left lung weight to body weight ratio ± standard error of the mean (SEM) were calculated from each litter of 8 ± 3 different pups and their corresponding left lung specimens in each group and at each time point in gestation. These results were then graphed using GraphPad Prism 7. qPCR results obtained via relative quantification were analyzed using the Student’s t-test to determine a difference in relative gene expression between oil and nitrofen groups across all time points in gestation and graphed using GraphPad Prism7. Corrected total cell fluorescence and western blot quantification results from immunohistochemistry were summarized with mean ± standard error of the mean (SEM). These results were then assessed for differences among all four groups (oil, nitrofen, nitrofen + IA dextrose, nitrofen + IA sildenafil) with one-way ANOVA with post-hoc Tukey HSD test. P-values < 0.05 were considered statistically significant.

## Electronic supplementary material

Supplementary Informatuon
