# Supplementary material for: Intra-amniotic Sildenafil Treatment Modulates Vascular Smooth Muscle Cell Phenotype in the Nitrofen Model of Congenital Diaphragmatic Hernia
Source: Sci Rep. 2018 Dec 5;8:17668. doi: 10.1038/s41598-018-34948-w (PMC6281652; doi:10.1038/s41598-018-34948-w)
Supplement: Supplementary file 1 — Supplementary Informatuon [file 41598_2018_34948_MOESM1_ESM.pdf]

Intra-amniotic Sildenafil Treatment Modulates Vascular Smooth Muscle Cell Phenotype in the Nitrofen Model of Congenital Diaphragmatic Hernia

Frances C. Okolo; Guangfeng Zhang; Julie Rhodes; Douglas A. Potoka

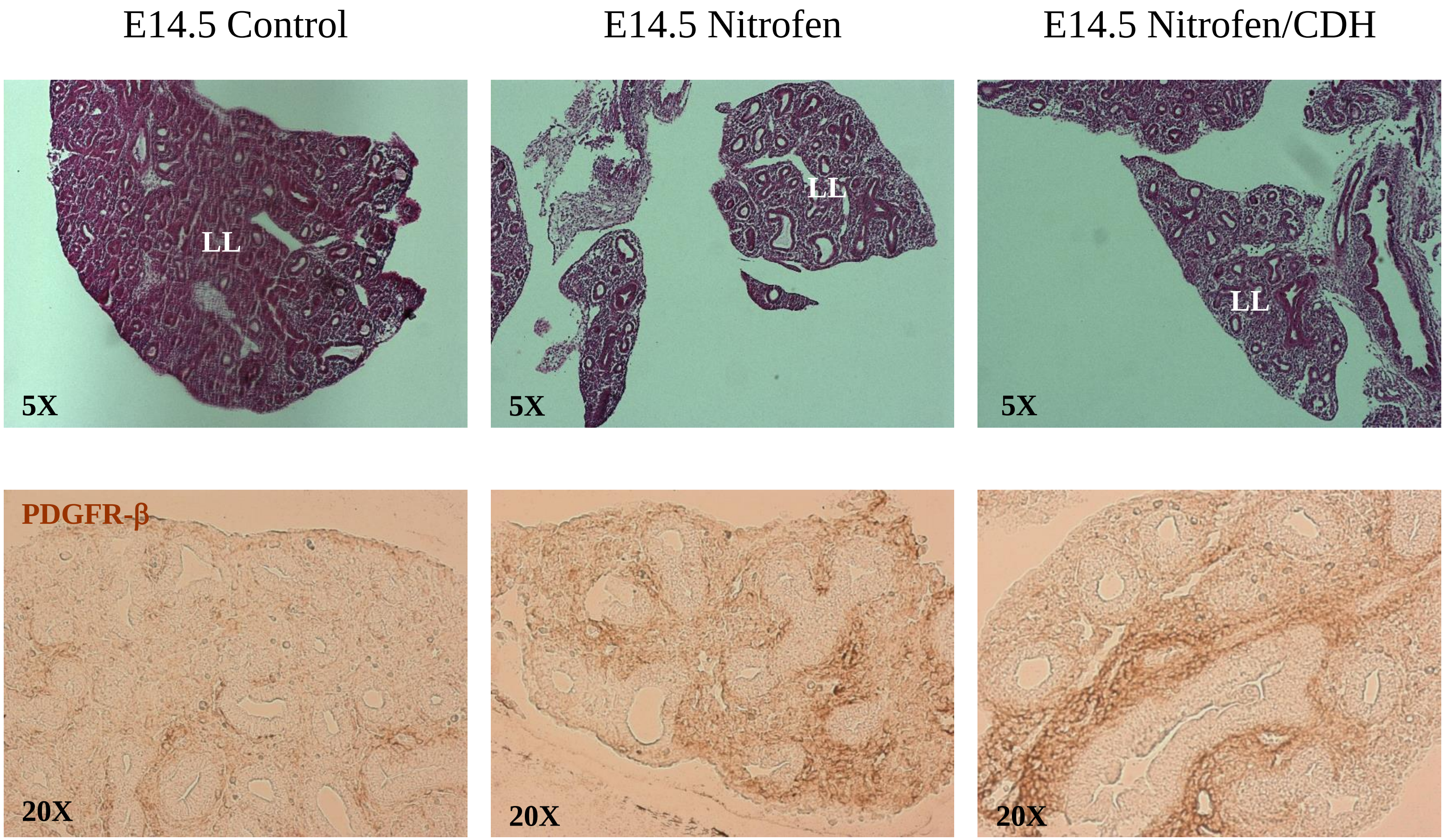

Supplementary Figure 1. H&E staining and PDGFR-β immunofluorescent staining pattern of the left lobe of the lung at E14.5 in oil, nitrofen, and nitrofen with CDH groups. The lung hypoplasia as well as the increased mesenchymal PDGFR-β expression in the nitrofen group is similar to that seen in the nitrofen with CDH group.

Primary Antibodies	Host	Manufacturer	Dilutions
Human/Mouse E-Cadherin Antibody	Goat	R&D Systems	1:100
Anti-Mouse CD31	Rat	BD Pharmingen	1:50
Anti-Desmin antibody	Rabbit	Abcam	1:300
Calponin 1/2/3	Rabbit	Santa Cruz Biotechnology, INC.	1:100
Anti-Myosin, Smooth muscle heavy chain 1 and 2 antibody	Rabbit	Abcam	1:500
Anti-alpha Smooth Muscle Actin antibody	Rabbit	Abcam	1:200
Anti-HES5 antibody	Rabbit	Abcam	1:1000
Mouse Notch-3 Antibody	Goat	R&D Systems	1:300
PDGF Receptor β mAb	Rabbit	Cell Signaling Technology	1:100
**Secondary Antibodies**			
Alexa Fluor 488 conjugated AffiniPure Anti-Rabbit IgG	Donkey	Jackson ImmunoResearch	1:200
Alexa Fluor 488 conjugated AffiniPure Anti-Mouse IgG	Donkey	Jackson ImmunoResearch	1:200
Cy3 conjugated AffiniPure F(ab')2 Fragment Anti-Rat IgG	Donkey	Jackson ImmunoResearch	1:200
Cy3 conjugated AffiniPure Anti-Rat IgG	Donkey	Jackson ImmunoResearch	1:200
Alexa Fluor 647 conjugated Anti-Goat IgG	Donkey	Jackson ImmunoResearch	1:200

Supplementary Table 1. Primary antibodies used for immunohistochemical staining with the corresponding secondary antibodies. Alexa Fluor 488 produces a green color, cy3 produces a red color and Alexa Fluor 647 produces a blue color.

Primary Antibodies	Host	Manufacturer	Dilutions
Anti-Desmin antibody	Rabbit	Abcam	1:500
Calponin 1/2/3	Rabbit	Santa Cruz Biotechnology, INC.	1:200
Anti-Myosin, Smooth muscle heavy chain 1 and 2 antibody	Rabbit	Abcam	1:1000
Anti-alpha Smooth Muscle Actin antibody	Rabbit	Abcam	1:1000
Anti-HES5 antibody	Rabbit	Abcam	1:150
Mouse Notch-3 Antibody	Goat	R&D Systems	1:150
PDGF Receptor β mAb	Rabbit	Cell Signaling Technology	1:1000
**Secondary Antibodies**			
Polyclonal Anti-Rabbit Ig/HRP	Goat	Dako	1:2000
Polyclonal Anti-Mouse Ig/HRP	Goat	Dako	1:1000
Polyclonal Anti-Goat Ig/HRP	Rabbit	Dako	1:1000

Supplementary Table 2. Primary antibodies used for western blot with the corresponding secondary antibodies. All rabbit derived antibodies were counterstained with anti-Rb Ig/HRP. All goat derived antibodies were counterstained with anti-Gt Ig/HRP. All mouse derived antibodies were counterstained with anti-Ms Ig/HRP.
